# Suspension culture of stem cells established of *Calendula officinalis* L.

**DOI:** 10.1038/s41598-023-50945-0

**Published:** 2024-01-03

**Authors:** Šarlota Kaňuková, Klaudia Lenkavská, Marcela Gubišová, Ján Kraic

**Affiliations:** 1 Department of Biotechnology, Faculty of Natural Sciences, University of Ss. Cyril and Methodius, Námestie J. Herdu 2, 917 01 Trnava, Slovakia; 2 Research Institute of Plant Production, National Agricultural and Food Center, Bratislavská cesta 122, 921 68 Piešťany, Slovakia

**Keywords:** Biological techniques, Biotechnology, Cell biology, Plant sciences, Stem cells

## Abstract

Plant stem cell cultures have so far been established in only a few plant species using cambial meristematic cells. The presence of stem cells or stem cell-like cells in other organs and tissues of the plant body, as well as the possibility of de novo generation of meristematic cells from differentiated cells, allow to consider the establishment of stem cell cultures in a broader range of species. This study aimed to establish a stem cell culture of the medicinal plant *Calendula officinalis* L. Callus tissues were induced from leaf and root explants, and already at this stage, stem and dedifferentiated cells could be identified. The cell suspension cultures established both from the root- and leaf-derived calli contained a high proportion of stem cells (92–93% and 72–73%, respectively). The most effective combination of growth regulators for the development of stem cells in calli as well as cell cultures was 1.0 mg/L 2,4-D and 0.5 mg/L BAP. The highest amount of stem cells (5.60–5.72 × 10^5^) was in cell suspension derived from the roots. An effective protocol for the establishment of marigold stem cell suspension culture was developed. The ratio of root-derived stem cells against dedifferentiated cells exceeded 90%.

## Introduction

*Calendula officinalis* L. (calendula), commonly known as the pot marigold or marigold, is cultivated as an ornamental annual or naturally grown as a perennial flowering plant of the family *Asteraceae*, genus *Calendula*. It has yellow or orange-colored edible florets that are also used as a dye in the production of foods and cosmetics. However, more interesting are the various pharmacological properties and therapeutic potentials attributed to flowers and leaves^1^. Calendula possesses many phytochemicals and pharmacological activities to be considered an excellent source of new drugs^2^. Many of these bioactive substances find application as antioxidants in cosmetics^3^. Extracts from calendula protect cells against UV radiation, stimulate skin cell regeneration, and improve skin elasticity^4^. In addition, anti-inflammatory, antimicrobial, immunostimulatory, as well as spasmolytic, hypolipidemic, antidiabetic, cardioprotective, hepatoregenerative, pancreas-regenerating, neuroprotective, anticancer, and other health-promoting effects, determine its applications in dermatology and medicine^5^. The Cosmetic Ingredient Review Expert Panel concluded that all ingredients from calendula are safe for applications in cosmetics under current practices^6^. Because of its interesting phytochemical composition, the traditional field growing of calendula plants is being replaced by the in vitro production of calendula cells and extracts from those cells as active cosmetic ingredients^7^.

The in vitro cultivation systems in calendula can produce plantlets by micropropagation^8^, but callus, cell suspension, and hairy root cultures can provide a continuous supply of calendula bioactive metabolites^7,9,10^. The induction of callus and its growth are fundamental steps for the establishment and applications of other plant tissue cultures. Callus tissues themselves can be used to prepare extracts with properties like antibiotics^11^, establish cell suspensions^12^, and protoplast cultures^13^. Hairy root cultures of calendula, induced by genetic transformation with *Agrobacterium rhizogenes*, can also effectively produce secondary metabolites^10,14–16^.

Different factors are responsible for the induction of callus in calendula, including genotype, explant type, culture medium, exogenous growth hormones, and others^17–19^. These factors affect not only the callus induction itself but also the growth parameters, morphogenetic capacity, and production potential of the callus. Cultivated calli and cell suspension cultures derived from calli are currently the most used platforms for in vitro biosynthesis of complex molecules or natural products, especially those with high economic value^20^. These in vitro production systems exploit mainly dedifferentiated cells developed from the differentiated cells of initial explants or, most often, a heterogeneous mixture of different types of cells. This approach has several limitations due to cellular heterogeneity and genetic and epigenetic instability. This is manifested by the slow growth rate of dedifferentiated plant cells, the lower yield of secondary metabolites, the aggregation of cells in the bioreactor, and mainly by undesirable variation in these parameters^21^. To overcome these problems and bypass the dedifferentiation step, a new platform in plant cell cultures has been described. It tries to use the potential of innately undifferentiated cambial meristematic cells (CMCs)^22^. CMCs may provide a robust, cost-effective, environmentally friendly platform and a sustainable source of plant cells and plant-derived natural products^23^. CMCs have plant stem cell properties^24^. They can actively divide, leading to the formation of different cells that eventually go through a differentiation process, and at the same time, produce new stem cells^25^. Populations of stem cells can be considered immortal. They are theoretically able to divide an unlimited number of times^26^ without being adversely limited by plant source, location, harvest period, or prevailing environmental conditions. The growth of stem cells (SCs) or stem cell-like cells (SCLCs) in culture in vitro is far superior to that of dedifferentiated cells in solid format (callus culture) as well as in liquid format (suspension culture)^26^. Moreover, SCs and SCLCs in suspension culture can tolerate shear stress, and the presence of small and abundant vacuoles avoids or reduces cell aggregation in bioreactors^27^.

To date, several studies on CMCs have been reported, but only in a limited number of plant species, mainly medicinal ones, e.g., *Taxus cuspidata*, *Ginkgo biloba*, *Solanum lycopersicon*^22^, *Panax ginseng*^22,28^, *Catharanthus roseus*^29^, *Tripterygium wilfordii*^30^, *Camptotheca acuminate*^31^, *Ocimum basilicum*^32^, *Fraxinus mandshurica*^33^. However, no study has been presented on the establishment and cultivation of stem cell or stem-like cell suspension cultures derived from *Calendula officinalis* L. Establishment of stem cell cultures in small volumes or in bioreactors should ensure higher production of cells and secondary metabolites in comparison with the cultivation of dedifferentiated cells in suspension cultures. Therefore, the aims of this work were to: (1) induce and characterize callus cultures; (2) establish cell suspension culture and determine its basic parameters; and (3) establish and characterize a line of stem cells or stem cell-like cells from *Calendula officinalis* L.

## Results

### Callus formation

The frequency of callus formation from leaf explants during the first 4 weeks was 100% using all combinations of auxin and cytokinin in the callus induction medium. The root explants also developed callus tissue at 100% frequency, but only in media containing 2,4-D as auxin. Lower frequencies (67–92%, rounded) were in media containing IAA as auxin (Table 1). Callus formation on leaf explants had already been initiated earlier, 12 days after they were placed on the induction medium. Root explants formed calli after 15 days. Callus formation after 4 weeks occurred along the entire length of root segments, while in leaf segments, calli were visible only on the cutting wounds, but later calli overgrew the whole leaf explant. Significant differences (*p* < 0.05) in the percentage of callus formation were observed between the leaf- and root-derived explants.

**Table 1:** Effects of explant types and plant growth regulators on the induction and growth of callus biomass of *Calendula officinalis* L.

Explant type	Plant growth regulators		Callus induction (%)		Callus weight (g)		Callus structure		Color of callus	
	Auxin (1 mg/L)	Cytokinin (0.5 mg/L)	4 weeks	8 weeks	4 weeks	8 weeks	4 weeks	8 weeks	4 weeks	8 weeks
Leaf	2.4-D	BAP	100	100	2.225 ± 0.208^B^	7.304 ± 2.200^A^	C	C	Yellow, light brown	Light brown, orange, yellow, gray
		2iP	100	100	2.315 ± 0.142^B^	7.577 ± 1.619^A^	C	WSF	Light yellow, orange	Yellow, orange, light/dark brown, black
		KIN	100	100	2.137 ± 0.096^B^	5.346 ± 1.007^A^	C	C	Light yellow, orange	Yellow, orange, light brown
		TDZ	100	100	1.907 ± 0.312^B^	6.302 ± 0.729^A^	C	C	Light yellow, orange	Yellow, orange, brown, black
	IAA	BAP	100	100	2.444 ± 0.935^B^	13.894 ± 2.588^A^	C	C	White, light brown	Light yellow/brown, orange
		2iP	100	100	6.066 ± 4.258^A^	9.911 ± 3.725^A^	C	WSF	Light yellow/orange, white	Orange, brown, white
		KIN	100	100	1.239 ± 0.142^B^	4.754 ± 0.540^A^	C	C	White, light orange/brown	White, yellow, orange, brown
		TDZ	100	100	3.110 ± 1.038^B^	8.150 ± 0.543^A^	C	C	Light yellow/orange, white	Light yellow/orange/brown, dark brown
Root	2.4-D	BAP	100	100	2.570 ± 1.159^ABC^	7.094 ± 2.019^A^	C	C	Yellow, light brown	Yellow, brown, orange, black
		2iP	100	100	3.345 ± 0.776^AB^	7.748 ± 1.995^A^	C	WSF/C	Light yellow, brown, orange	White, gray, yellow, light/dark brown, orange, black
		KIN	100	100	3.581 ± 0.322^A^	6.910 ± 1.135^A^	C	C	Yellow, brown	Yellow, orange, light/dark brown, black
		TDZ	100	100	2.129 ± 0.559^CD^	6.711 ± 3.046^A^	C	C	Yellow, light brown	Yellow, orange, brown, black
	IAA	BAP	83.33	91.67	1.418 ± 0.643^DE^	7.256 ± 1.576^A^	C	C	Light yellow	Light yellow/brown, orange
		2iP	91.67	100	2.397 ± 0.711^BCD^	8.082 ± 2.167^A^	C	WSF	Light yellow	White, yellow, light/dark brown, orange
		KIN	66.67	66.67	0.803 ± 0.374^E^	3.371 ± 1.612^B^	C	C	Light yellow/orange/brown	Yellow, orange, light/dark brown
		TDZ	91.67	91.67	3.190 ± 0.185^ABC^	9.476 ± 0.374^A^	C	C	Light yellow	Light yellow/brown, dark brown, orange, black

Explant type	Plant growth regulators		Callus induction (%)		Callus weight (g)		Callus structure		Color of callus	
	Auxin (1 mg/L)	Cytokinin (0.5 mg/L)	12 weeks	16 weeks	12 weeks	16 weeks	12 weeks	16 weeks	12 weeks	16 weeks
Leaf	2.4-D	BAP	100	100	12.474 ± 2.828^B^	16.907 ± 2.189^B^	C	CS	Yellow, light brown, gray	Black, brown, white, gray, orange
		2iP	100	100	11.549 ± 0.855^BC^	12.858 ± 3.117^CD^	WSF	WSF	Yellow, orange, light/dark brown, black	Black, brown, orange
		KIN	100	100	8.826 ± 1.482^CD^	12.489 ± 2.406^CD^	C	C	Yellow, orange, brown	Black, brown, orange
		TDZ	100	100	10.210 ± 1.030^BCD^	13.455 ± 1.765^CD^	C	CS	Yellow, orange, brown, black	Orange, brown, black
	IAA	BAP	100	100	19.027 ± 1.785^A^	22.866 ± 1.574^A^	WS	WSF	Orange, brown	Orange, light/dark brown, white
		2iP	100	100	10.912 ± 3.771^BC^	14.069 ± 1.979^BC^	WSF	C	Brown, white	Brown, light/dark yellow
		KIN	100	100	7.226 ± 0.115^D^	10.671 ± 0.488^D^	C	C	White, yellow, orange, brown, black, gray	White, yellow, orange, brown, black, gray
		TDZ	100	100	10.553 ± 1.480^BCD^	15.078 ± 0.265^BC^	C	CS	Yellow, orange, gray, lihgt/dark brown	Orange, gray, brown, black
Root	2.4-D	BAP	100	100	11.650 ± 2.667^AB^	13.433 ± 3.305^BCDE^	C	CS	Yellow, orange, brown, black	Orange, brown, black
		2iP	100	100	11.066 ± 2.441^AB^	12.141 ± 2.819^DE^	WSF/C	WSF, SC	Yellow, ligh/dark brow, orange, black	Brown, black, orange
		KIN	100	100	9.937 ± 1.546^BC^	12.280 ± 1.351^CDE^	C	C	Yellow, orange, light/dark brown, black	Black, brown, orange
		TDZ	100	100	11.573 ± 4.056^AB^	15.521 ± 2.627^BC^	C	CS	Yellow, orange, brown, black, gray	Yellow, orange, brown, black
	IAA	BAP	91,67	91,67	11.858 ± 1.696^AB^	16.204 ± 3.934^AB^	WS	WSF	Yellow, orange, brown	Light yellow, orange, brown, white
		2iP	100	100	9.809 ± 1.240^BC^	15.014 ± 3.753^BCD^	WSF	C	White, yellow, orange, brown	White, light/dark yellow, orange, brown
		KIN	66,67	66,67	6.722 ± 4.237^C^	9.620 ± 4.237^E^	C	C	Yellow, orange, brown, white	Yellow, orange, brown, white
		TDZ	91,67	91,67	13.550 ± 0.617^A^	15.446 ± 3.184^B^	C	CS	Yellow, orange, light/dark brown, black, gray	Orange, brown, black, gray

Callus structure: *C* compact, *WS* watery soft, *WSF* watery soft and friable, *2,4-D* 2,4-dichlorophenoxyacetic acid, *IAA* indole-3-acetic acid, *BAP* 6-benzylaminopurine, *2iP* 6-(γ,γ-dimethylallylamino)purine, *KIN* kinetin, *TDZ* thiadiazuron (1-phenyl-3-(1,2,3-thiadiazol-5-yl)urea). Exponents^A,B,C,D,E^ indicate significant different average values (p < 0.05).

The color of the calli that developed after 4 weeks varied according to the combinations of auxin and cytokinin used. The calli were multicolored. However, lighter colors prevailed, mainly yellow. During subsequent cultivation, the color gradually changed. After 16 weeks of cultivation (i.e., after three passages), most of them partially or completely darkened to brown and black, and necrotization was also observed. The structure of a callus usually relates to its quality, regarding the processes of morphogenesis and plant regeneration. After 4 weeks of cultivation, all calli induced both from leaf and root explants, regardless of combinations of growth regulators, were compact. The compact callus had a firm texture that could not be easily cut. But, already after the first and subsequent passages, its structure changed to watery soft and watery soft friable, especially if the 2iP was used as a cytokinin (Table 1).

Some combinations of growth regulators in the medium also induced the development of roots, already after 4 weeks of cultivation. This was observed in the combinations IAA + 2iP and IAA + KIN in leaf explants, and IAA + 2iP and IAA + TDZ in root explants.

### Callus growth

**Figure 1:**
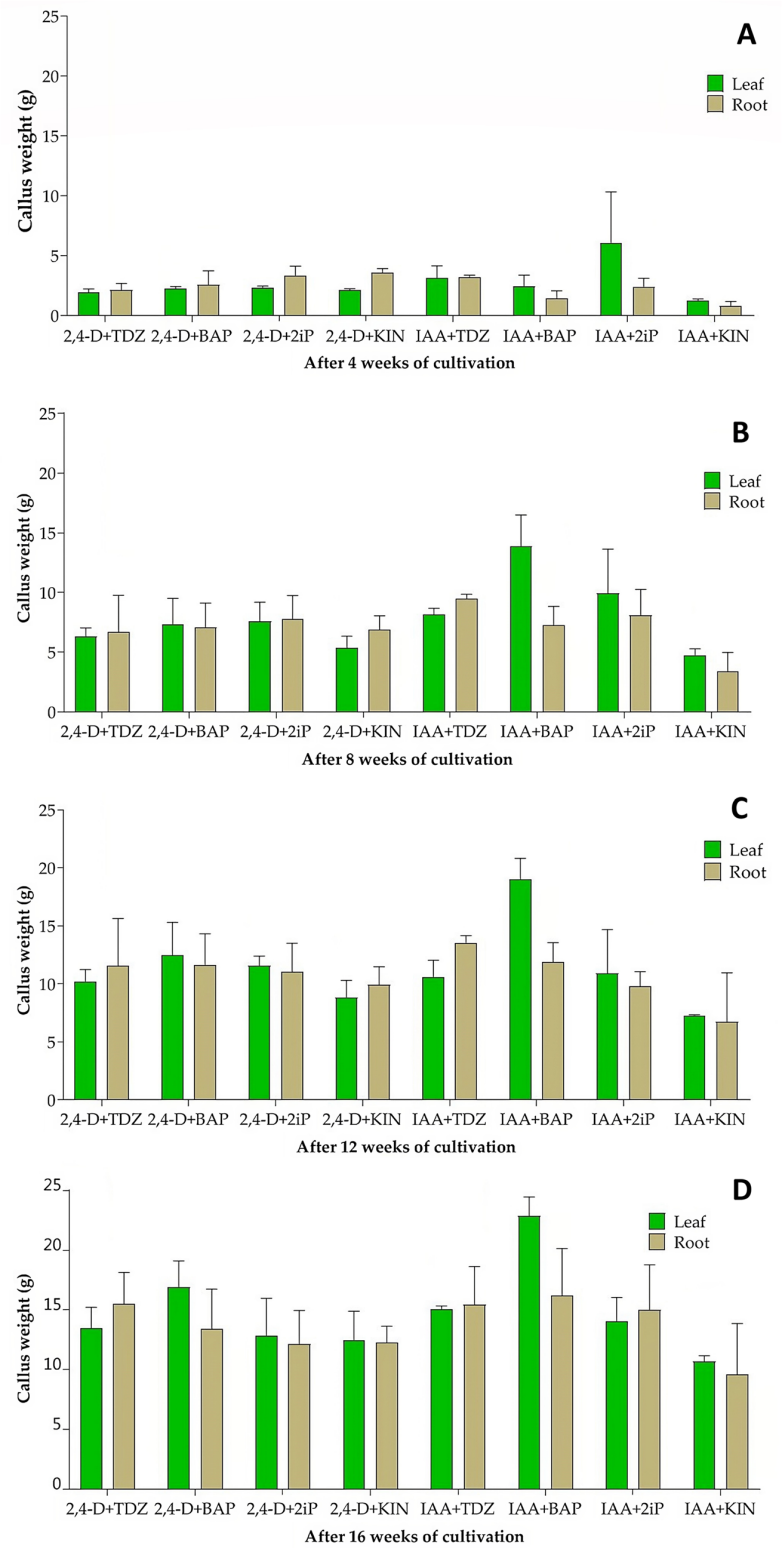
Fresh weight of calli induced from leaf and root explants using different combinations of growth regulators after four (**A**), eight (**B**), 12 (**C**), and 16 (**D**) weeks of cultivation. Data are presented as means ± SDs (n = 5). Indicators of the statistical significance of the difference between combinations of growth regulators are shown in Table 1 (exponents^A,B,C,D^).

The highest average fresh weight of callus biomass derived from leaves (6.07 g) after 4 weeks of cultivation was on the medium with the combination IAA + 2iP (Fig. 1A). Unfortunately, these calluses developed roots, which undesirably increased their weight. Moreover, after 8, 12, and 16 weeks, this combination of growth regulators no longer produced the highest callus biomass weight. For this reason, this combination of growth regulators was excluded from the following experiments aimed at analyzing the presence of stem cells. The average fresh weight of callus biomass derived from leaves, using all other combinations of growth regulators, ranged from 1.24 g (IAA + KIN) to 3.11 g (IAA + TDZ) and callus biomass derived from root explants was in the range of 0.80–3.58 g (Fig. 1A). The increase in fresh weight of callus biomass continued for all combinations of growth regulators after the first and second passages (Fig. 1B,C). However, the most important growth parameters of callus biomass were growth intensity and fresh weight determined after three subsequent passages (i.e., after 16 weeks), always on fresh medium. The continuous increase in the fresh weight of callus biomass continued until the 16th week of cultivation in media with all combinations of growth regulators and with both types of explants (Fig. 1D).

**Figure 2:**
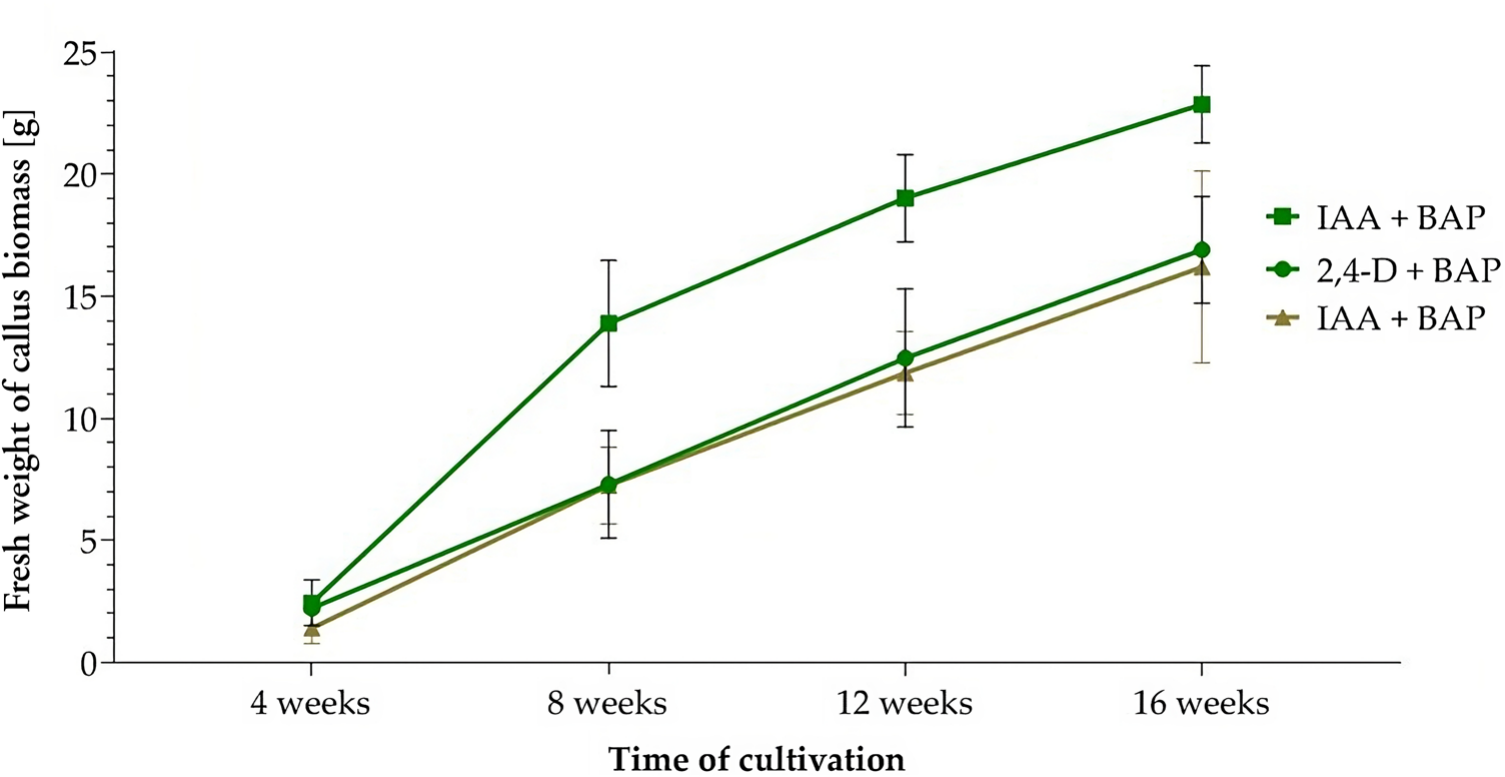
Increase in fresh weight of callus biomass derived from leaf (green curves) and root (brown curve) explants within 16 weeks of cultivation on the most effective combinations of growth regulators.

The highest increase in fresh biomass weight after 16 weeks of cultivation was in calli derived from leaf tissue in medium containing combinations of IAA + BAP (from 2.44 to 22.87 g) and 2,4-D + BAP (from 2.23 to 16.91 g). Also, in the case of callus biomass derived from roots, the same combination of IAA + BAP (from 1.42 to 16.20 g) was the most effective. The intensity of the increase in the fresh weight of the callus biomass was linear over the monitored period (Fig. 2).

### Stem cells in callus

**Figure 3:**
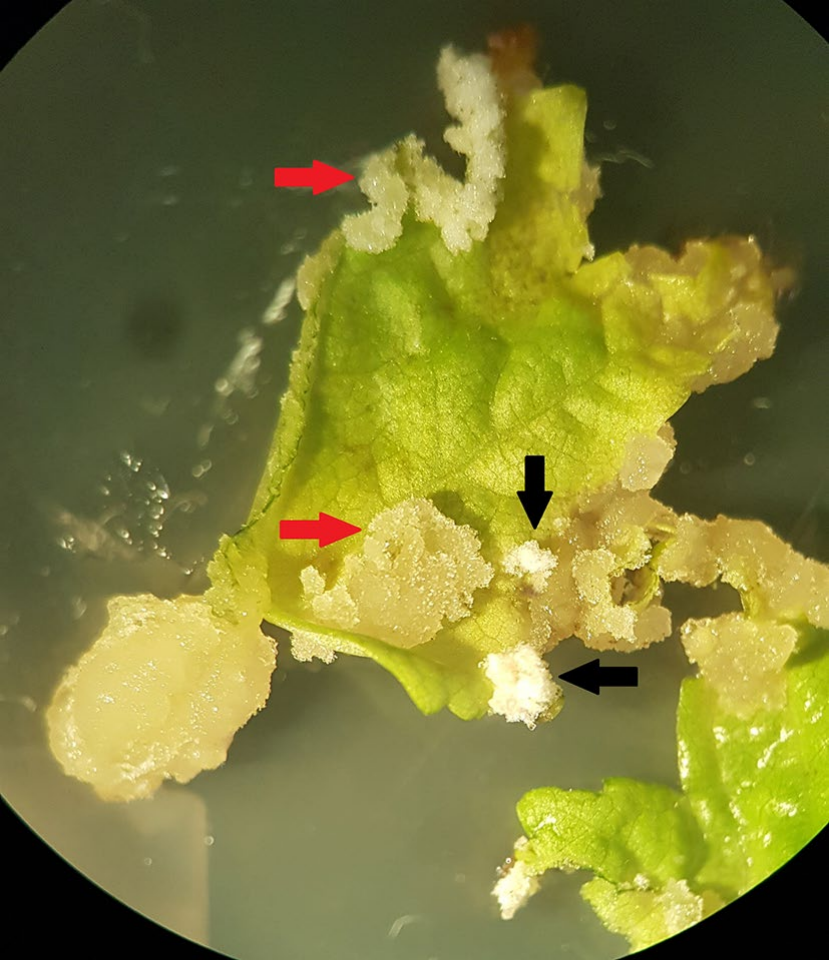
Different types of calli (color, texture) on the same leaf explant (red arrows: stem cells, black arrows: dedifferentiated cells, magnification ×6.3).

Cells with the properties of stem cells or stem cell-like cells should be present in fast-growing multicellular callus biomass, along with dedifferentiated and differentiated cells. The presence of SCs or cells similar to them in the growing cell biomass should be supported by the morphological features of cells developed on the callus surface. After 4 weeks of cultivation, different types of callus cell biomass, containing SCs, cells similar to them (stem cell-like cells), as well as DDCs (dedifferentiated cells), could be visually observed. Calli with potentially proliferating SCs were light yellow, dim, and soft, while DDCs were light white, compact, and had an irregular structure (Fig. 3). However, these parameters can be ambiguous as they may vary depending on the plant species, the explant, and the callus induction conditions.

**Figure 4:**
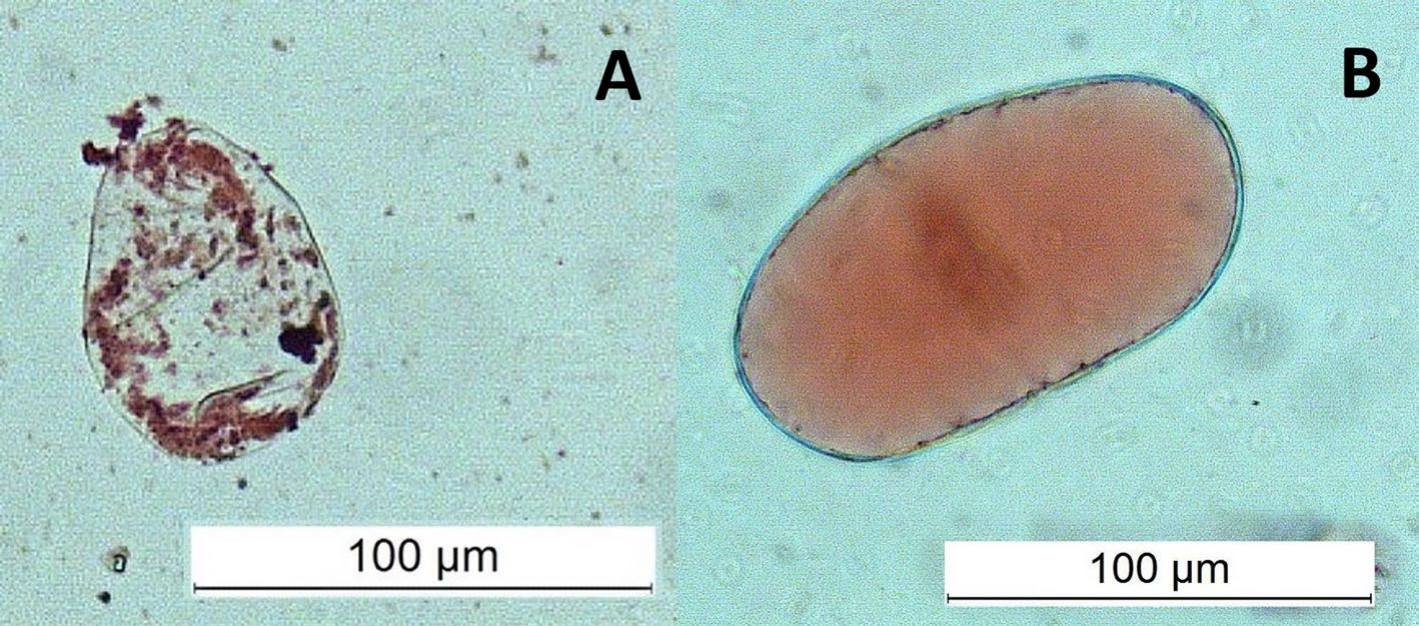
Abundant and very small vacuoles in stem cells (**A**) and one large vacuole in dedifferentiated cells (**B**).

Microscopic analysis revealed typical differences in the morphology of vacuoles between SCs and DDCs stained with the Neutral Red assay. Abundant and small spheric vacuoles, or vacuole-like structures, were observed in SCs (Fig. 4A), while only one large vacuole was present in DDCs (Fig. 4B). These differences in the parameters of the vacuoles are considered characteristic.

**Figure 5:**
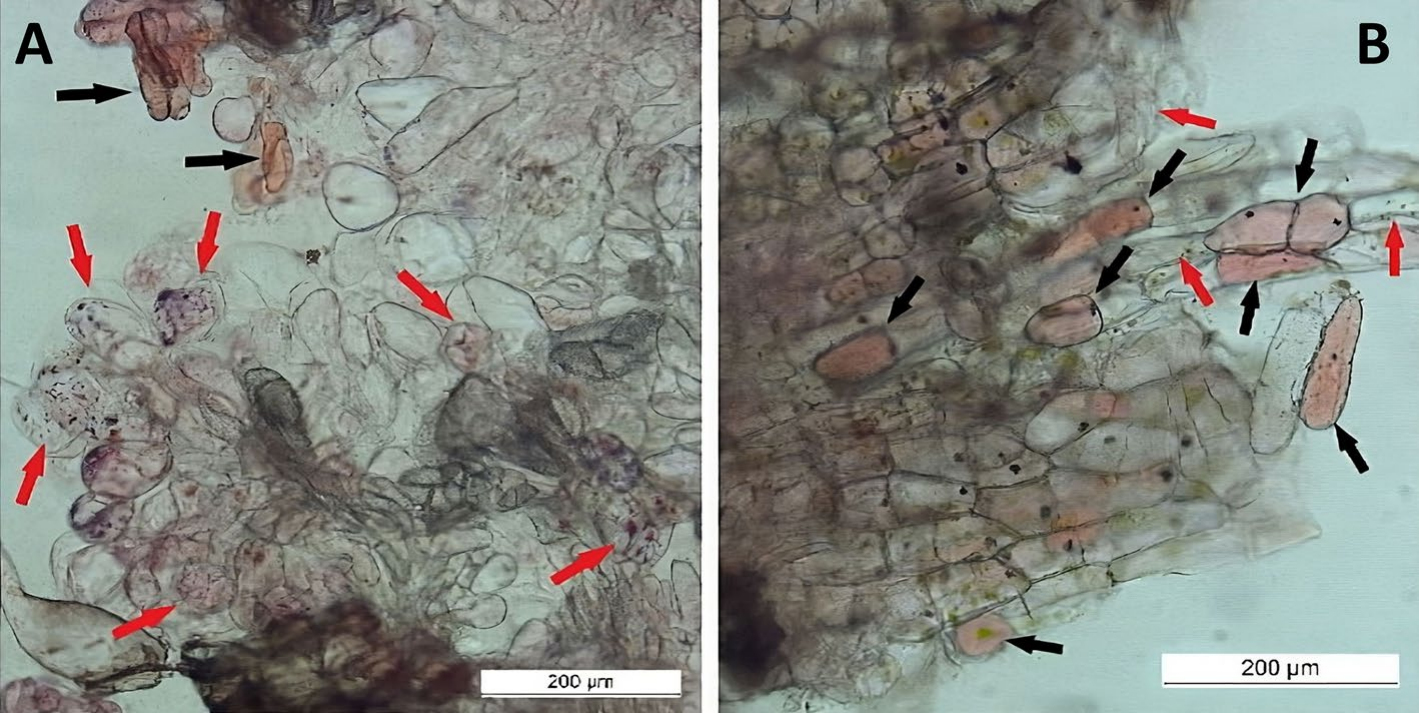
Stem cells (red arrows), dedifferentiated cells (black arrows), and other cells in cell biomass derived from leaf (**A**) or root (**B**) explants formed in medium containing 1.0 mg/L 2,4-D + 0.5 mg/L BAP.

The relative ratio between SCs and other cells in the growing cell biomass was determined subjectively using microscopic observations. The differences in ratios between SCs and DDCs were related to the explant source. A higher proportion of SCs was subjectively evaluated in callus biomass derived from leaves (Fig. 5A), while callus derived from root explants contained a higher proportion of DDCs and other cell types (Fig. 5B).

**Figure 6:**
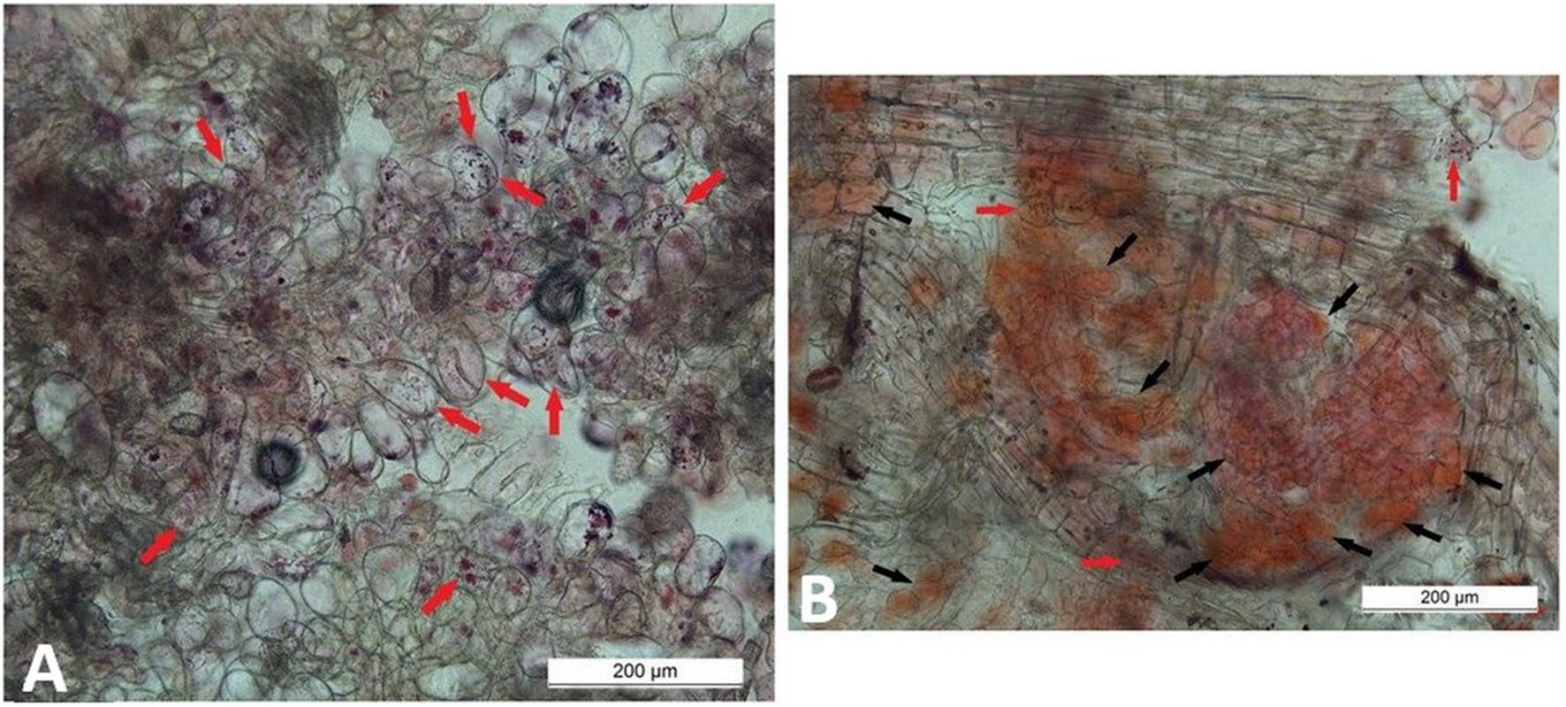
Stem cells (red arrows) and dedifferentiated cells (black arrows) in callus biomass derived from leaf and cultivated in MS medium with 1.0 mg/L 2,4-D + 0.5 mg/L BAP (**A**) or 1.0 mg/L IAA + 0.5 mg/L BAP (**B**).

Two combinations of growth regulators that most effectively promoted the growth of leaf-derived callus cell biomass were subjectively compared in terms of the number of SCs. Apparently, higher relative amounts of SCs over DDCs were found in medium containing 1.0 mg/L 2,4-D with 0.5 mg/L BAP (Fig. 6A). On the contrary, the combination of 1.0 mg/L IAA with 0.5 mg/L BAP generated predominantly DDCs (Fig. 6B).

### Cell suspension cultures

**Figure 7:**
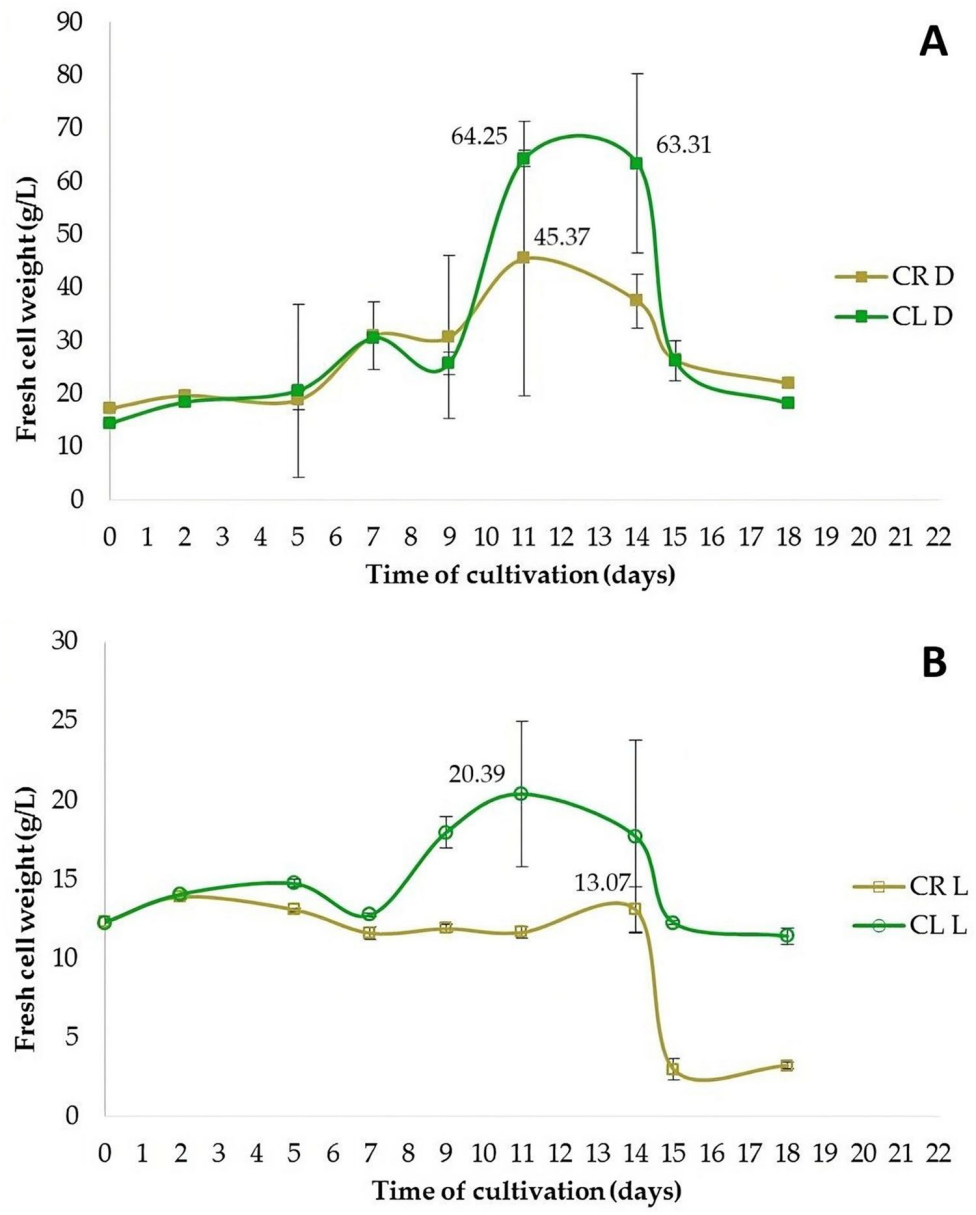
Fresh weight of cells (g/L) in suspension culture originated from leaf-derived (CL) and root-derived (CR) callus, cultivated in medium MS with 1.0 mg/L 2,4-D + 0.5 mg/L BAP continuous darkness (**A**) or under the photoperiod (**B**).

Cell suspension cultures were derived from callus tissues induced either from leaf or root explants in the same composition of nutrient medium, either in continuous darkness or under photoperiod (16 h light/8 h darkness). The growth characteristics of cells in suspensions were different (Fig. 7). The highest fresh cell weight (FCW, g/L) was achieved between the 11th and 13th days after the inoculation of cells into the fresh liquid MS medium with 1.0 mg/L 2,4-D + 0.5 mg/L BAP. The optimal interval for passaging of cells into fresh liquid medium was 12–13 days (Fig. 7A) in cell suspensions cultivated in darkness and 11 days in cell suspensions cultivated under the photoperiod. The numerical values shown in Fig. 7 show when the maximum fresh cell weight was reached. Cultivation in continuous darkness produced 3.8 times more root-derived cells and 3.2 times more leaf-derived cells in comparison to cultivation under the photoperiod (Fig. 7B). Therefore, only the darkness regime of cultivation was used in the following experiments. Statistically significant (p < 0.05), the highest production of fresh cells (64.3 g of cells/L of cultivation medium) was achieved in cell suspension derived from leaves. It was 1.42 times higher than in root-derived cell suspension.

**Figure 8:**
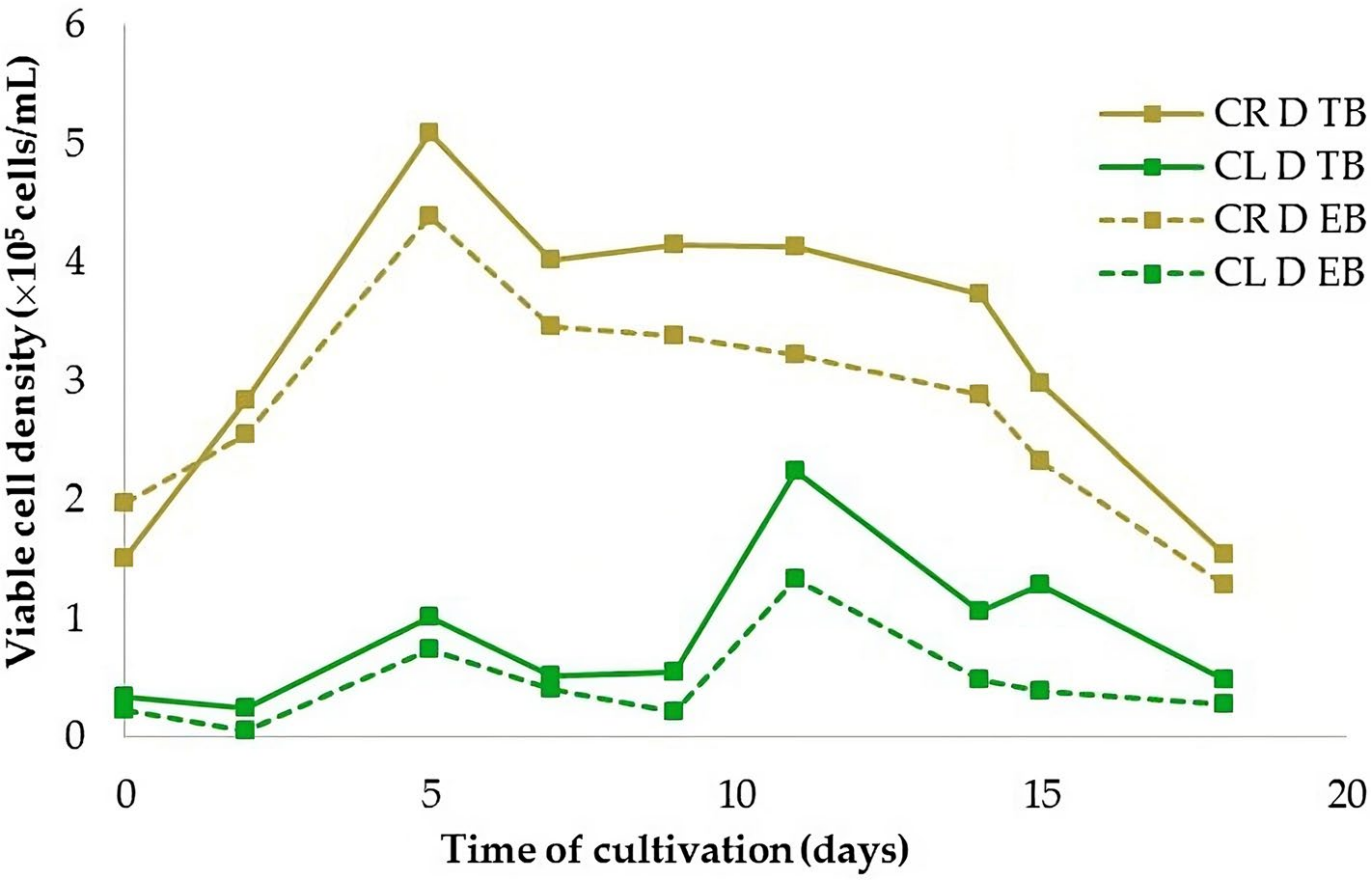
The viability of cells in leaf-derived (CL D) and root-derived (CR D) suspension cultures cultivated in MS medium with 1.0 mg/L 2,4-D + 0.5 mg/L BAP continuous darkness assayed by Evans blue (EB) and Trypan blue (TB).

Determination of cell viability revealed that cell suspensions developed from root-derived callus (CR) were more viable than those from leaf-derived callus (CL). The maximum number of viable root-derived cells (5.08 × 10^5^ cells/mL) was reached on the fifth day after the initiation of cell suspension culture. In leaf-derived cells, the maximum number of viable cells was lower by 56% (2.24 × 10^5^ cells/mL) and reached later, on the 11th day of cultivation. It has been shown that both stains, Evans blue and Trypan blue, are essentially equally appropriate for cell viability detection (Fig. 8).

### Stem cell suspension cultures

**Figure 9:**
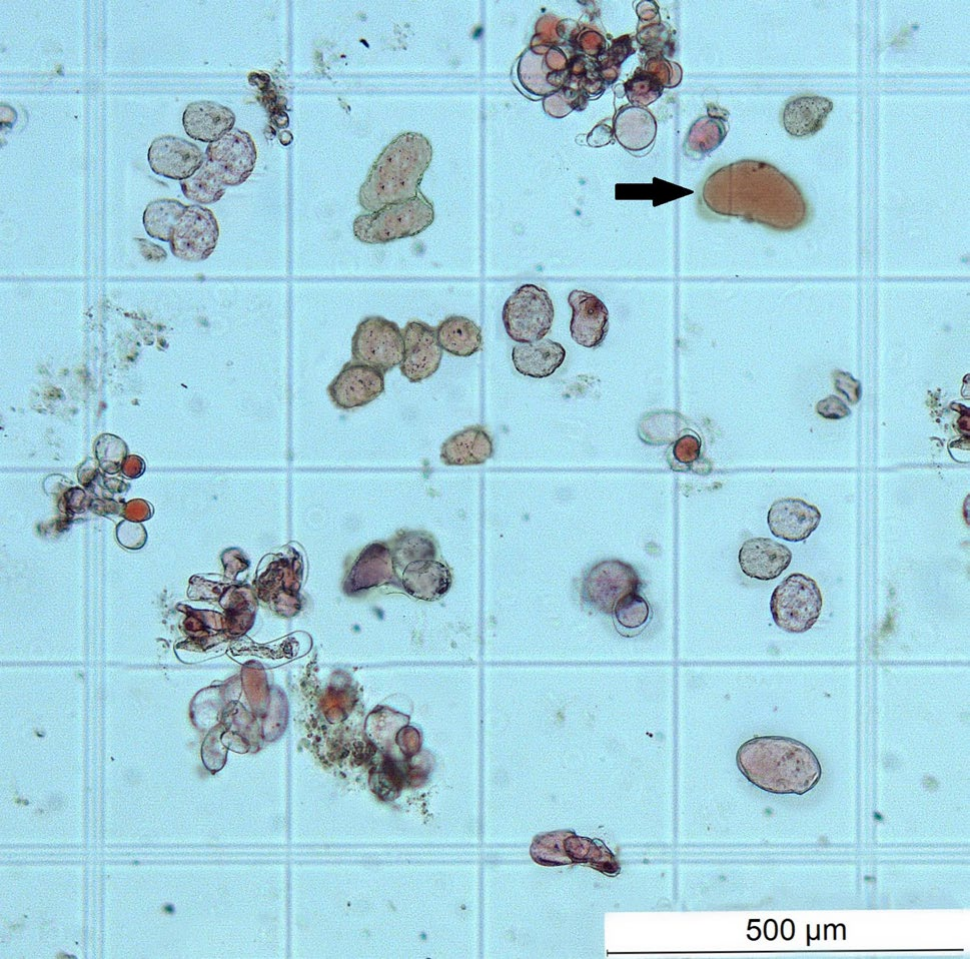
Stem cells and small amounts of dedifferentiated cells (black arrow) in stem cell suspension culture established from root-derived callus and cultivated in liquid MS medium with 1.0 mg/L 2,4-D + 0.5 mg/L BAP.

The cell suspension cultures were used for the selection and establishment of a culture of stem cells, i.e., a culture with the highest possible proportion of SCs versus DDCs. The Neutral Red assay was much easier in cell suspension compared to callus tissue. SCs had typical, very small, and abundant vacuoles. Prolonged cultivation time and repeated passages of cells into fresh liquid medium eliminated or fundamentally reduced the aggregation of cells, and the single-cell character of the cell suspension began to dominate. At the same time, the fewer aggregates and more individual cells were in the cell suspension, the higher the proportion of stem cells (Fig. 9).

**Figure 10:**
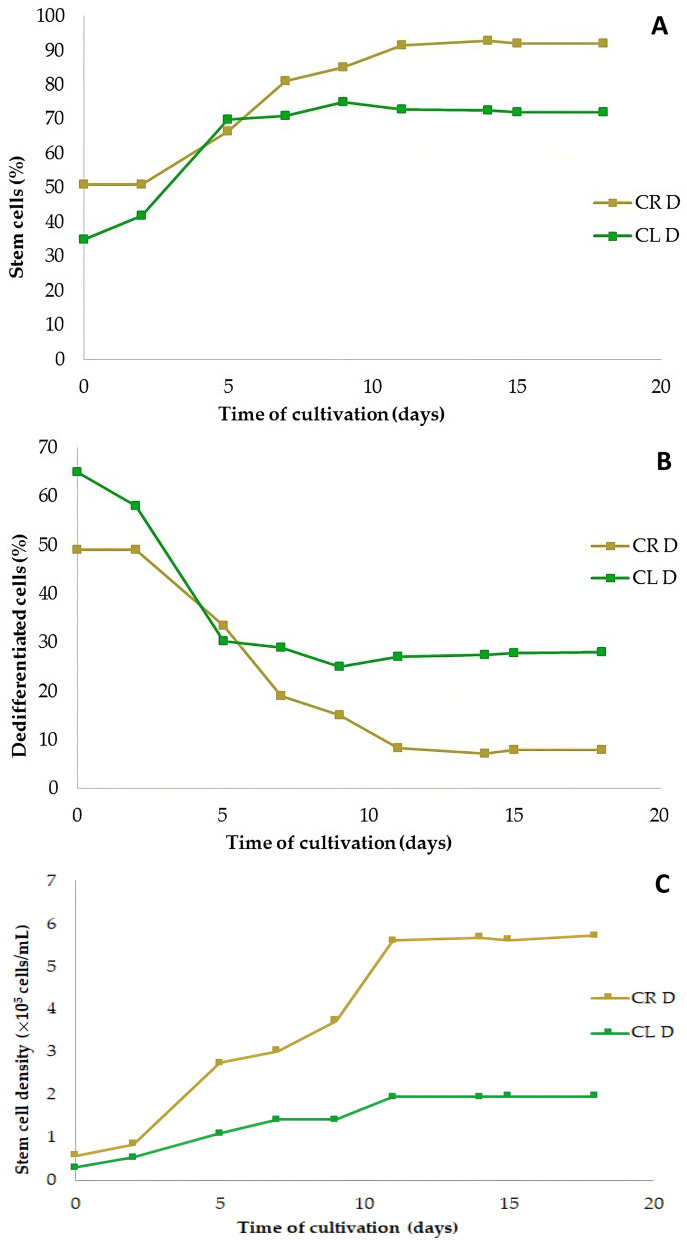
Proportion of stem cells (**A**) and dedifferentiated cells (**B**) in cell suspension cultures derived from leaves (CL D) and roots (CR D). The number of stem cells (**C**) derived from roots (CR D) and leaves (CL D). Culture medium was MS with 1.0 mg/L 2,4-D + 0.5 mg/L BAP in all cultures, cultivation was in continuous darkness.

The ratio of SCs to DDCs in suspension culture had opposite tendencies. As the ratio of SCs increased (Fig. 10A), the ratio of DDCs decreased adequately (Fig. 10B). The ratio of SCs derived from roots exceeded the level of 91.6% on the 11th day and was maintained in the range of 92–93%, in contrast to root-derived DDCs that gradually decreased to less than 8%. A similar course had a proportion of leaf-derived SCs that reached a maximum also on the 9th day, but only at the level of 75.0%, and subsequently it stabilized at the level of 72–73%. The share of leaf-derived DDCs decreased adequately to 27–28%. The number and percentage of SCs did not decrease at all, even on the 18th day of cultivation.

From the point of view of the number of SCs produced, root cells were more productive than leaf cells (Fig. 10C). Their multiplication reached a plateau on the 11th day of cultivation, and the number of SCs then stabilized at a level of 5.60–5.72 × 10^5^ cells/mL. The growth of leaf-derived stem cells also reached a plateau on the 11th day of cultivation at a level of 2.2 × 10^5^ cells/mL i.e., about 2.5 times less.

## Discussion

The plant species, type of explant, and composition of the cultivation medium, especially the content of growth regulators, are well-known crucial factors affecting the initiation of callus development and growth in vitro. All combinations of auxins and cytokinins used in our experiments were able to induce callogenesis in both types of *C. officinalis* L. explants, the leaf as well as the root segments. In the presented work, the most effective combination of auxin and cytokinin was IAA + BAP. The combinations 2,4-D + KIN and 2,4-D + 2iP used previously^12^ induced calli with much lower efficiency. Other growth hormones have proven effective for other explants, such as hypocotyls, cotyledons, cotyledon nodes^11^, and floret explants^35^. Calli initiated from different types of explants of *C. officinalis* L. tend to be variable in their color and texture, and it also change during cultivation depending on the growth regulators used for callus initiation. The coloration of the calli in this study ranged from white to yellow, orange, brown, and black, and they were often multicolored. Similar coloration and texture were also found in calli derived from hypocotyls^11^, but the calli derived from the leaves and cotyledons were in shades of green^12^. Generally, callus cultures of calendula can be easily induced from different types of explants using culture media containing a mixture of auxin and cytokinin. Calli differ in their texture, from firm and compact to friable and watery. Moreover, sometimes they are very heterogeneous in these characteristics.

Callus culture can also be used for the micropropagation of calendula plants^8,36^. The long-term cultivation and multiplication of callus from calendula, either unelicited or elicited, can be used for the production of useful compounds such as carotenoid pigments^35^, salicylic acid^37^, and extracts with antibacterial activity^11^. The callus induction stage is already important for the subsequent establishment of cell suspension culture. From the point of view of callus disintegration, a fine cell suspension culture in a liquid medium with a friable structure of callus is more advantageous. The character of cells in callus is especially important in establishing a fast-growing, long-term cultivable, and more productive cell suspension cultures. The callus is initiated either from explants containing meristems with pluripotent stem cells or from explants containing essentially only somatic cells. Callus is formed predominantly from a pre-existing population of stem cells^38^ and from dedifferentiated cells arising through transformation from differentiated cells. Dedifferentiated cells are in a state of less differentiation or in a stem-like state (stem cell-like cells), which gives them the properties of totipotency^39,40^. If stem cells are already present in the explant used for callus induction and appropriate growth regulators are used, the callus biomass typically has improved growth parameters^22,33^. The presumed presence of calendula stem cells was experimentally confirmed by microscopic analyses of calluses that had the fastest and largest increase in callus biomass. The effect of these cells was also reflected in the parameters of the cell suspension cultures derived from them, such as rapid cell growth rate, exponential increase in fresh cell weight, lower aggregation rate, cell size and shape, and others^22,30,32,33^. Homogenization of the cell suspension culture to single-cell culture was achieved by several passages of cells into fresh culture medium in the late exponential phase, which was approximately on the 13th day after initiation. An increased homogeneity of the suspension culture toward the single-cell culture correlated with the increasing proportion of SCs versus DDCs in the culture. Plant stem cell cultures have so far been established exclusively from isolated and cultured innately undifferentiated cambial meristematic cells from stem segments^22,29–34^. However, the use of cambial meristematic cells from stem explants may not be the only way to establish in vitro stem cell cultures. A callus resembling the root tip meristem can be developed from pericycle cells of mature roots that retain some meristematic cell-specific features, and this is also possible from cells surrounding vasculature bundles in other aerial organs^41^. Meristematic features of some pericycle cells, such as three or more vacuoles and dense cytoplasm^42^ are considered typical features of plant stem cells. Also, leaves maintain their own meristems. Leaf mesophyll cells, as well as the initial cells of stomata and veins, are produced by leaf meristems classified as intercalary meristems^43^. The leaf meristems contain the plate meristem, which extends the leaf two-dimensionally by active cell proliferation^44^ as well as the marginal meristem restricted to the leaf margin^45^. The plate and marginal meristems, both active in the basal area of leaf primordia, represent two zones of a leaf meristem, analogous to the central and peripheral zones of the shoot apical meristem^46^. Leaves exhibit determinate growth, indicating that the potential leaf meristem, if it exists, has only transient meristematic activity, but the leaf marginal and submarginal regions maintain active cell divisions during early leaf development and are responsible for leaf lamina initiation^47^. Other studies suggested that marginal meristem activity contributes little to the growth of the leaf blade, but the blade growth in angiosperm is sustained by cell proliferation in a plate meristem region^44^.

Another possible way is related to de novo organogenesis and plant regeneration in plant tissue cultures. Plant somatic cells can, after reprogramming, form a mass of pluripotent cells in auxin-induced callus tissue^48^. These pluripotent and regeneration-competent cells in the root are termed vasculature-associated pluripotent cells, and the callus originates from xylem-pole pericycle and pericycle-like cells^49^. The regeneration-competent cells were found in leaf explants, throughout the mature leaf along the vasculature in dicot plants, and in the leaf base in monocot plants^50^. Just cells acquiring pluripotency are crucial for the formation of the callus and the promeristem, and finally for de novo shoot regeneration by indirect organogenesis^48,51^. Shoots and roots can be regenerated through de novo-formed meristems^52^ and by gene editing after the concomitant expression of developmental regulators and gene-editing reagents^53^. Thus, the use of explants derived from roots and leaves provides a prerequisite for the possibility of establishing stem and stem cell cultures.

The latest findings change opinions not only about the nature and composition of callus tissues but also about meristems, stem cells, and the processes of their de novo formation. Views on the capabilities of differentiated cells and the processes of their dedifferentiation are also changing. This opens the way not only to the isolation of plant stem cells but also to their propagation, the establishment of in vitro stem cell cultures, the production of stem cell biomass, and the more efficient production of secondary metabolites. This opens interesting industrial and economic perspectives for plant biotechnology, tissue and cell cultures, and their use to obtain additional theoretical knowledge as well as practical applications. An overview of several metabolites from different plant species, produced by several types of in vitro culture systems, confirms the existence of such applications. It also demonstrates that stem cells are much more efficient producers than cultures of dedifferentiated cells^34^. Cell cultures in vitro are a robust, cost-effective, and sustainable alternative for large-scale production of desired metabolites compared to traditional plant cultivation.

## Conclusions

Meristematic cells contained in primary explants or formed de novo in tissue cultures can determine the establishment of high-effective production of cell biomass through callus tissue and cell suspension culture. However, much more interesting and important is the possibility to establish the formation or to select stem cells and cultivate them in a liquid medium as a cell suspension with a very high proportion of stem cells. In the presented work, a culture of stem cells was established from segments of the leaves and roots of *C. officinalis* L. In the case of this medicinal plant, it seems that the way to establish stem cell culture is neither impossible nor too difficult. The successful establishment of stem cell cultures, their long-term cultivation in small and medium-sized bioreactors, and elicitation of cell biomass and metabolites production will lead to practical applications in some medical fields, cosmetics, and elsewhere.

## Methods

### Plant material

Seeds of *Calendula officinalis* L. were obtained from the breeding company Zelseed spol. s r. o. (Horná Potôň, Slovakia). Surface sterilization of mature seeds was performed with 96% ethanol (v/v) for 30 s, followed by treatment with a 4.7% (v/v) sodium hypochlorite solution for 5 min with constant stirring and rinsing five times with sterile distilled water. Seeds were germinated in vitro in culture vessels containing MS medium^54^ (Duchefa Biochemie B.V, Haarlem, Netherlands) with a reduced concentration of all components (1/2MS) and 0.8% agar (w/v), adjusted to pH 5.7. Germinated seeds and seedlings were cultivated in growth room at 23 ± 2 °C under a photoperiod of 16 h of light and 8 h of darkness.

In vitro experiments with plants complied with accordance with the relevant institutional and national legislation.

### Callus cultures

Explants were taken from the leaves and roots of 4–5-week-old aseptic plants. The leaf explants (middle part of leaf blade, without leaf base and tip) were approximately 0.5 × 0.5 cm in size, and the length of the root explants was approximately 0.5 cm. The culture medium used was MS medium^53^ which contained 30 g/L (w/v) sucrose, 8 g/L (w/v) of plain agar powder, and complete nutrients in the form of macro, micro, trace elements, and vitamins, supplemented with plant growth hormones and regulators, respectively. Combinations of two auxins: 2,4-D or IAA with four cytokinins –2iP, KIN, BAP, or TDZ in a ratio 2: 1 (1 mg/L auxin and 0.5 mg/L cytokinin) were tested for callus induction (Table 1). Twenty-five explants have been used for each combination of plant growth regulators. Cultures were incubated at 23 ± 2 °C in the dark and sub-cultured at 28-day intervals on the fresh medium for four months. The fresh weight of the callus biomass was determined under sterile conditions as the total fresh weight of the same number of calli grown on individual combinations of growth hormones after 4, 8, 12, and 16 weeks, respectively.

### Cell suspension cultures

Cell suspension cultures were derived from calli developed from leaf and root explants in the liquid MS medium supplemented with 1.0 mg/L 2,4-D + 0.5 mg/L BAP. Cultivation was at the same temperature (23 ± 2 °C) either in darkness or under the photoperiod (16 h of light and 8 h of darkness) with a light intensity of 50 μmol m^−2^ s^−1^. Cells were cultivated under constant stirring (VS-202P, Vision Scientific Co., Ltd., Daejeon, Korea) at 110 rpm in 100 ml Erlenmayer flasks containing 20 ml of liquid medium. The optimal sub-cultivation interval was identified by observation of the cell growth rate by regular cell samplings during 18 days of cultivation and determination of fresh cell weight and dry cell weight.

The fresh weight of cells was calculated by weighing the cell residue on filter paper after filtering the entire volume of the flask and subtracting the weight of the filter paper. The dry weight was determined by drying the filtered fresh cells in an oven at 65 °C for 2 h.

Cell viability was determined by staining procedures using two stains. Cell suspension was mixed with a 0.1% (w/v) solution of Evan’s blue in a ratio 5:1 or with a 0.4% solution of Trypan blue (both from Merck KGaA, Darmstadt, Germany) in a ratio 1:1. Viable or dead cells were detected microscopically (Leica DM6000 Upright Optical Microscope, Leica Microsystems GmbH, Wetzlar, Germany). Observations were performed in three replicates.

### Stem cell cultures

Microscopic analysis of cells was performed in developed callus tissues by dyeing vacuoles using the modified method of Lee et al.^22^. Cells taken from callus biomass were stained with 0.01% (w/v) Neutral Red (3-amino-7-dimethyl-amino-2-methylphenazine hydrochloride, Merck KGaA, Darmstadt, Germany) for 10 min and washed with 0.1 M phosphate buffer, pH 7.2. Samples were prepared by the squash smear technique and observed using the Leica DM6000 Upright Optical Microscope (Leica Microsystems GmbH, Wetzlar, Germany).

Stem cells in suspension culture were identified by the same method as in callus cultures. However, cell suspension was mixed with Neutral Red in a ratio 1:1, centrifuged for 5 min at 2500 rpm, washed with 0.1 M phosphate buffer, pH 7.2, and counted in the Fuchs-Rosenthal Counting Chamber (Paul Marienfeld GmbH & Co. KG, Lauda-Königshofen, Germany) under the same microscope. The experiments were performed in three replicates.

### Data analysis

The effects of growth regulators used for callus induction and growth were analysed in five replications, each with five leaf- or root-derived explants, respectively. Evaluated parameters included the day of the initial callus formation, the fresh weight of the callus, the percentage of callus formation, as well as the morphology parameters, including the color and structure of the callus. Obtained data were evaluated by analysis of variance (one-way ANOVA) followed by the least significant difference (LSD) test using Statgraphics software version 19.2.01 (Statgraphics Technologies, Inc., The Plains, VA, USA). Significant differences between means were compared using the least significant difference (LSD) test at the 5% level of significance (*p* < 0.05).

## Data Availability

All data generated or analysed during this study are included in this published article.

## Abbreviations

CMC: Cambial meristematic cells
SCLCs: Stem cell-like cells
DDCs: Dedifferentiated cells
MS: Murashige and Skoog basal medium
1/2MS: Murashige and Skoog with half concentration of all components
2,4-D: 2,4-Dichlorophenoxyacetic acid
IAA: Indole-3-acetic acid
2iP: 6-(γ,γ-Dimethylallylamino)purine
KIN: Kinetin
BAP: 6-Benzylaminopurine
TDZ: Thidiazuron
SD: Standard deviation
FCW: Fresh cell weight

## References

[1] Ashwlayan VD, Kumar A, Verma M, Garg VK, Gupta SK (2018). Therapeutic potential of *Calendula officinalis*. Pharm. Pharmacol. Int. J..

[2] Jan N, Andrabi KI, John R (2017). *Calenula officinalis*—an important medicinal plant with potential biological properties. Proc. Indian Natl. Sci. Acad..

[3] Xuan SH, Kim GY, Yu JY, Kim JW, Yang YR, Jeon YH (2016). Antioxidant and cellular protective effects against oxidative stress of *Calendula officinalis* flowers extracts in human skin cells. Appl. Chem. Eng..

[4] Akhtar N, Zaman SU, Khan BA, Amir MN, Ebrahimzadeh MA (2011). Calendula extract: Effects on mechanical parameters of human skin. Acta Pol. Pharm..

[5] Szopa A, Klimek-Szczykutowicz M, Jafernik K, Koc K, Ekiert H (2020). Pot marigold (*Calendula officinalis* L.)—a position in classical phytotherapy and newly documented activities. Acta Sci. Pol. Hortorum Cultus.

[6] Andersen FA, Bergfeld WF, Belsito DV, Hill RA, Klaassen CD, Liebler DC (2010). Final report of the Cosmetic Ingredient Review expert panel amended safety assessment of *Calendula officinalis*-derived cosmetic ingredients. Int. J. Toxicol..

[7] Georgiev V, Slavov A, Vasileva I, Pavlov A (2018). Plant cell culture as emerging technology for production of active cosmetic ingredients. Eng. Life Sci..

[8] Çöçü S, Uranbey S, Ipek A, Khawar KM, Sarihan EO, Kaya MD (2004). Adventitious shoot regeneration and micropropagation in *Calendula officinalis* L.. Biol. Plant..

[9] Wiktorowska E, Dlugosz M, Janiszowska W (2010). Significant enhancement of oleanolic acid accumulation by biotic elicitors in cell suspension cultures of *Calendula officinalis* L.. Enzyme Microb. Technol..

[10] Długosz M, Wiktorowska E, Wiśniewska A, Pączkowski C (2013). Production of oleanolic acid glycosides by hairy root established cultures of *Calendula officinalis* L.. Acta Biochim. Pol..

[11] Çetin B, Kalyoncu F, Kurtuluş B (2017). Antibacterial activities of *Calendula officinalis* callus extract. Int. J. Sec. Metab..

[12] Grzelak A, Janiszowska W (2002). Initiation and growth characteristics of suspension cultures of *Calendula officinalis* cells. Plant Cell Tissue Organ. Cult..

[13] Auguścińska E, Kasprzyk Z (1982). Studies on the labelling of terpenoids in shoots and cells or protoplasts from *Calendula officinalis* leaves. Acta Biochim. Pol..

[14] Długosz M, Markowski M, Pączkowski C (2018). Source of nitrogen as a factor limiting saponin production by hairy root and suspension cultures of *Calendula officinalis* L.. Acta Physiol. Plant..

[15] Alsoufi ASM, Pączkowski C, Szakiel A, Długosz M (2019). Effect of jasmonic acid and chitosan on triterpenoid production in *Calendula officinalis* hairy root cultures. Phytochem. Lett..

[16] Rogowska A, Paczkowski C, Szakiel A (2022). Modulation of steroid and triterpenoid metabolism in *Calendula officinalis* plants and hairy root cultures exposed to cadmium stress. Int. J. Mol. Sci..

[17] Mehrabi AA, Khodadadi E, Sadeghi Z, Shooshtari L (2013). An investigation of tissue culture and co-cultures of different explants in *Calendula officinalis*. Int. J. Biosci..

[18] Kaya N, Aki C (2017). In vitro effects of plant growth regulators on callus formation in *Calendula officinalis* L. and *Calendula arvensis* L. species. Ann. Biol. Res..

[19] Al-Abasi IN, Bashi BZK, Al-Mallah MK (2018). Design of culture medium and leaf clones are determinant factors in callus induction of *Calendula officinalis* L.. Eur. Acad. Res..

[20] Efferth T (2019). Biotechnology applications of plant callus cultures. Engineering.

[21] Kolewe ME, Gaurav V, Roberts SC (2008). Pharmaceutically active natural product synthesis and supply via plant cell culture technology. Mol. Pharm..

[22] Lee EK, Jin YW, Park JH, Yoo YM, Hong SM, Amir R (2010). Cultured cambial meristematic cells as a source of plant natural products. Nat. Biotechnol..

[23] Ochoa-Villarreal M, Howat S, Jang MO, Kim IS, Jin Y-W, Lee E-K (2015). Cambial meristematic cells: A platform for the production of plant natural products. New Biotechnol..

[24] Ye ZH (2002). Vascular tissue differentiation and pattern formation in plants. Annu. Rev. Plant Biol..

[25] Laux T (2003). The stem cell concept in plants: A matter of debate. Cell.

[26] Yun BW, Yan Z, Amir R, Hong S, Jin YW, Lee EK (2012). Plant natural products: History, limitations and the potential of cambial meristematic cells. Biotechnol. Genet. Eng. Rev..

[27] Joshi JB, Elias CB, Patole MS (1996). Role of hydrodynamic shear in the cultivation of animal, plant and microbial cells. Chem. Eng. J..

[28] Lee SB, Cho HI, Jin YW, Lee EK, Ahn JY, Lee SM (2016). Wild ginseng cambial meristematic cells ameliorate hepatic steatosis and mitochondrial dysfunction in high-fat diet-fed mice. J. Pharm. Pharmacol..

[29] Moon SH, Venkatesh J, Yu JW, Park SW (2015). Differential induction of meristematic stem cells of *Catharanthus roseus* and their characterization. C R Biol..

[30] Song Y, Chen S, Wang X, Zhang R, Tu L, Hu T (2019). A novel strategy to enhance terpenoids production using cambial meristematic cells of *Tripterygium wilfordii* Hook f.. Plant Methods.

[31] Zhang Y, Jiang J, Qin N, Zhang Q, Yan C (2019). Biotransformation of 4-methylcoumarins by cambial meristematic cells of *Camptotheca acuminate*. RSC Adv..

[32] Mehring A, Haffelder J, Chodorski J, Stiefelmaier J, Strieth D, Ulber R (2020). Establishment and triterpenoid production of *Ocimum basilicum* cambial meristematic cells. Plant Cell Tissue Organ Cult..

[33] He L, Zhang J, Guo D, Tian H, Cao Y, Ji X (2021). Establishment of the technology of cambial meristematic cells (CMCs) culture from shoots and high expression of *FmPHV* (*PHAVOLUTA*) functions in identification and differentiation of CMCs and promoting the shoot regeneration by hypocotyl in *Fraxinus mandshurica*. Plant Physiol. Biochem..

[34] Partap M, Warghat AR, Kumar S (2022). Cambial meristematic cell culture: A sustainable technology towards in vitro specialized metabolites production. Crit. Rev. Biotechnol..

[35] Legha MR, Prasad KV, Singh SK, Kaur C, Arora A, Kumar S (2012). Induction of carotenoid pigments in callus cultures of *Calendula officinalis* L. in response to nitrogen and sucrose levels. In Vitro Cell Dev. Biol. Plant.

[36] Leal, F., *et al*. In vitro multiplication of *Calendula arvensis* for secondary metabolites extraction. In *Proceedings of the IIIrd International Symposium on Acclimatization and Establishment of Micropropagated Plants, Faro, Portugal*, 28 February 2009.

[37] Ibrahim MM, Abed RM, Ali FQ (2019). Influence of biotic elicitor *Aspergillus niger* on salicylic acid products in callus cultures of *Calendula officinalis* L. plant. J. Phys. Conf. Ser..

[38] Sugimoto K, Gordon SP, Meyerowitz EM (2011). Regeneration in plants and animals: Dedifferentiation, transdifferentiation, or just differentiation?. Trends Cell Biol..

[39] Grafi G (2004). How cells dedifferentiate: A lesson from plants. Dev. Biol..

[40] Verdeil J-L, Alemanno L, Niemenak N, Tranbarger TJ (2007). Pluripotent versus totipotent plant stem cells: Dependance versus autonomy?. Trends Plant Sci..

[41] Sugimoto K, Jiao Y, Meyerowitz EM (2010). Arabidopsis regeneration from multiple tissues occurs via a root development pathway. Dev. Cell.

[42] Parizot B, Laplaze L, Ricaud L, Boucheron-Dubuisson E, Bayle V, Bonke M (2008). Diarch symmetry of the vascular bundle in Arabidopsis root encompasses the pericycle and is reflected in distich lateral root initiation. Plant Physiol..

[43] Ichihashi Y, Tsukaya H (2015). Behavior of leaf meristems and their modification. Front. Plant Sci..

[44] Donnelly PM, Bonetta D, Tsukaya H, Dengler RE, Dengler NG (1999). Cell cycling and cell enlargement in developing leaves of *Arabidopsis*. Dev. Biol..

[45] Maksymowych R, Erickson RO (1960). Development of the lamina in *Xanthium italicum* represented by the plastochron index. Am. J. Bot..

[46] Alvarez JP, Furumizu C, Efroni EY, Bowman JL (2016). Active suppression of a leaf meristem orchestrates determinate leaf growth. eLife.

[47] Du F, Guan C, Jiao Y (2018). Molecular mechanisms of leaf morphogenesis. Mol. Plant.

[48] Shin J, Bae S, Seo PJ (2020). De novo shoot organogenesis during plant regeneration. J. Exp. Bot..

[49] Atta R, Laurens L, Boucheron-Dubuisson E, Guivarc’h A, Carnero E, Giraudat-Pautot V (2009). Pluripotency of *Arabidopsis xylem* pericycle underlies shoot regeneration from root and hypocotyl explants grown in vitro. Plant J..

[50] Hu B, Qi M, Xu L, Zhang G, Li J, Qin P (2017). Divergent regeneration-competent cells adopt a common mechanism for callus initiation in angiosperms. Regeneration.

[51] Müller-Xing R, Xing Q (2022). The plant stem-cell niche and pluripotency: 15 years of an epigenetic perspective. Front. Plant Sci..

[52] Ikeuchi M, Favero DS, Sakamoto Y, Iwase A, Coleman D, Rymen B (2019). Molecular mechanisms of plant regeneration. Annu. Rev. Plant Biol..

[53] Maher MF, Nasti RA, Vollbrecht M, Starker CG, Clark MD, Voytas DF (2020). Plant gene editing through de novo induction of meristems. Nat. Biotechnol..

[54] Murashige T, Skoog F (1962). A revised medium for rapid growth and bioassays with tobacco tissue cultures. Physiol. Plant..

